# Online Handwritten Signature Verification Using Neural Network Classifier Based on Principal Component Analysis

**DOI:** 10.1155/2014/381469

**Published:** 2014-07-14

**Authors:** Vahab Iranmanesh, Sharifah Mumtazah Syed Ahmad, Wan Azizun Wan Adnan, Salman Yussof, Olasimbo Ayodeji Arigbabu, Fahad Layth Malallah

**Affiliations:** ^1^Department of Computer and Communication Systems Engineering, Universiti Putra Malaysia, 43400 Serdang, Selangor, Malaysia; ^2^Department of Systems and Networking, Universiti Tenaga Nasional, Jalan IKRAM-Uniten, 43000 Kajang, Malaysia

## Abstract

One of the main difficulties in designing online signature verification (OSV) system is to find the most distinctive features with high discriminating capabilities for the verification, particularly, with regard to the high variability which is inherent in genuine handwritten signatures, coupled with the possibility of skilled forgeries having close resemblance to the original counterparts. In this paper, we proposed a systematic approach to online signature verification through the use of multilayer perceptron (MLP) on a subset of principal component analysis (PCA) features. The proposed approach illustrates a feature selection technique on the usually discarded information from PCA computation, which can be significant in attaining reduced error rates. The experiment is performed using 4000 signature samples from SIGMA database, which yielded a false acceptance rate (FAR) of 7.4% and a false rejection rate (FRR) of 6.4%.

## 1. Introduction 

Biometrics can be literally described as human biological characteristics that can be used for recognition [1]. Biometric recognition systems are normally developed for two main purposes, which are identification and verification. The deployment of biometric computerized applications for providing access control and monitoring is now common in a variety of public organizations, financial institutes, and airports [2, 3]. A biometric system can be modeled based on either physical or behavioral traits of individuals [1]. Physical traits such as face, fingerprint, and iris are very unique to every individual and are stable over an extended period of time [1]. Hence, biometric systems, which are based on these traits, are usually accurate and reliable enough for identification purposes that involve one to many comparisons [1, 4].

On the other hand, behavioral traits such as voice, gait, and signature may be susceptible to changes over time [1] and can be skillfully mimicked by impostor [5]. Thus designing an accurate behavioral based biometric system is a challenging task.

While many biometric technologies suffer from privacy-intrusion issues, handwritten signature is perhaps a specific biometric trait that is widely accepted by the general public [2, 3]. This is mainly because of the long-dated history of signatures as tokens for verification of financial transactions and legal document bindings [2, 3, 6].

In an automated handwritten signature verification system, the collected biometric samples of a user's signature are usually stored in a database as reference templates to be used as basis for subsequent verification stages. However, intrauser variability, which is defined as changes in the genuine templates of the same user, is one of the greatest challenges in signature biometrics since it affects the accuracy of the system [7–10]. In addition, given sufficient signature samples, a forgery can be produced with a high degree of close resemblance to the original counterparts [8, 11, 12].

There are two main approaches to signature-based biometrics, namely, online and offline approaches [13]. In the offline approach, also known as the static approach, the signature image is scanned or captured using a camera or scanner after the signature is signed on a paper. On the other hand, the online (dynamic) technique is capable of extracting dynamic user features (trajectories, pressure, velocity, etc.) during the signing operation and captures the information using digitizing devices, such as tablet or touch pad [13]. This research work focuses on the latter approach, as it allows for a richer set of information to be captured in addition to the signature images.

**Figure 1:**
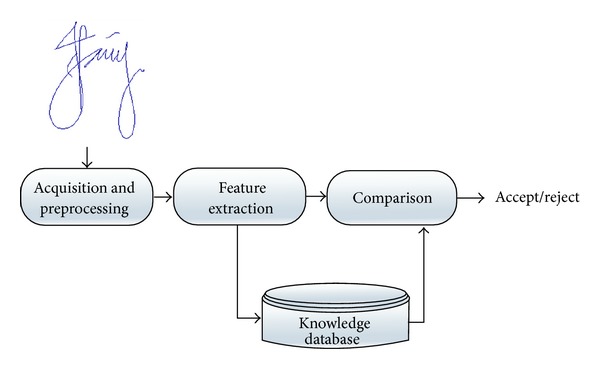
Online signature verification system schema.

Figure 1 depicts the basic structural design of an online signature verification (OSV) system [16]. Initially, signature samples are collected in the enrollment stage, whereby useful information known as dynamic features is extracted in order to build a user's reference template, which is stored in the knowledge database. Then, the template is used as a reference for comparison with the new queried user's features to decide either to reject or to accept the queried signature sample as genuine or not [17]. It is virtually impossible for a user to reproduce his/her exact signature on multiple attempts due to intrauser variability. Intrauser variability measures the difference between the signatures of an individual, which may be influenced by environmental, health, and emotional challenges while signing [14, 18].

In the last decade, a number of studies have been carried out on online and offline signature verification with the sole aim of improving the verification accuracy [19]. The verification system should also incorporate lesser computational complexity in order to provide fast response for real-time applications [16]. Several classification methods have been suggested for robust verification purposes, one of which includes artificial neural network (ANN) [20–24]. Thus, in this paper, we maintain the use of ANN as the classifier and focus on improvement at the feature level.

To this end, we propose the use of function-based features that provide more detailed signature dynamics compared to the conventional parameter features such as number of pen-ups and pen-downs and displacement [16]. In order to reduce the data dimension, principal component analysis (PCA) is used on the signature time series signals such as pen trajectories (*x*, *y*) and pen pressure (*p*).

**Figure 2:**
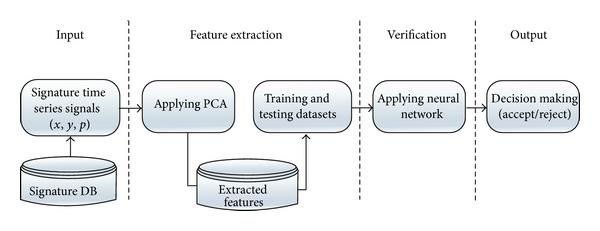
A schematic diagram of suggested online signature verification system.

The overview of the proposed architecture is shown in Figure 2. First, the time series signals (*x*, *y*, *p*) are extracted as PCA features, such as components, latents, and scores. Then, these features are used in training and testing stages based on a multilayer perceptron (MLP) classifier with 200 users and 8,000 samples to detect genuine or forged signatures.

The rest of this paper is structured as follows. The experimental signature database is described in Section 2.  Section 3 illustrates the materials and methods.  Section 4 evaluates the experimental results. We discuss our findings in Section 5. Finally, a conclusion is drawn in Section 6.

## 2. Experimental Handwritten Signature Database 

**Figure 3:**
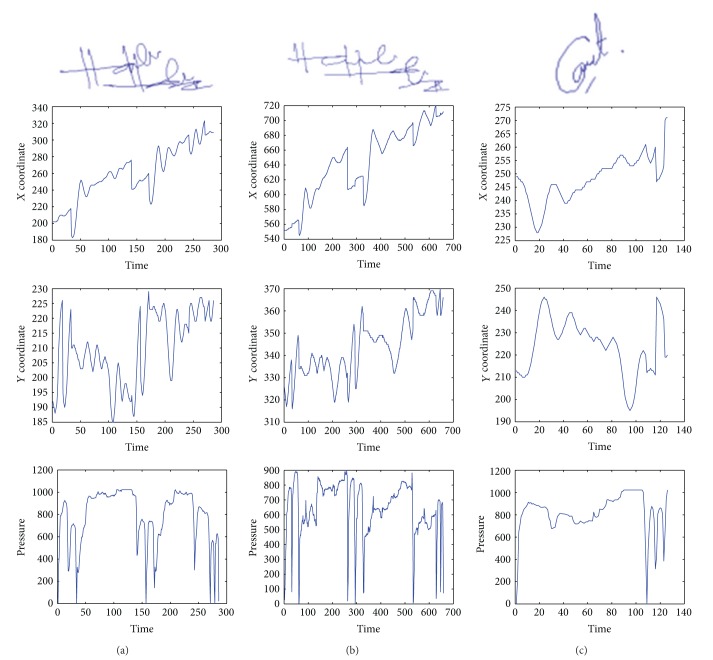
Sample of signature pen trajectories and pressures in SIGMA DB: (a) genuine signature sample; (b) skill-forged signature sample; and (c) user 193 genuine signature sample as a non-skill-forged signature.

The database used for this study is the SIGMA database [25]. A random subset of 200 users which is composed of 20 genuine, 10 skill-forged, and 10 non-skill-forged signatures for each user is selected. In the training phase, 10 genuine, 5 skill-forged, and 5 non-skill-forged signatures are selected to represent each user's signature sample in the training phase. Similarly, the same number of samples is used during the testing phase. A genuine signature is labeled 1, and a forged signature is labeled 0. The total signature samples selected for the training set are 4,000, and the remaining 4,000 samples are used in the testing set.  Table 1 summarizes the number of samples utilized in this study. The signatures in the mentioned database are represented by time series signals such as pen trajectories (*x*, *y*) and pen pressure (*p*) at each sampling point as shown in Figure 3.

## 3. Materials and Methods

In this study, PCA is used to analyze the signature time series signals to decrease the feature space dimensionality and extract new prominent features. Then we performed a strategic feature selection by selecting some other elements in PCA computation such as latent and score. Finally, the obtained features from the feature extraction and selection stages are combined to represent the signature at the classification stage.

### 3.1. Feature Extraction and Selection

PCA is one of the most popularly used statistical methods for feature extraction, dimension reduction, and data representation in pattern recognition and computer vision [26]. The basic concept of PCA involves mapping multidimensional data distribution into a lower dimension with reduced loss of important information. It is achieved by projecting the raw data with high correlation between variables to a new space with uncorrelated variables [24]. The resulting principal components are utilized as extracted features to represent the data.

As initially pointed out in Section 2, in our selected signature subset, each signature sample in the SIGMA database is composed of three time series signals (*x*, *y*, *p*), resulting in a feature vector with high dimensionality. However, in order to represent the feature space of each signature in a lower dimension, we consider six fundamental steps for computing PCA, before performing feature selection. The procedural steps are simplified as follows.

**Step 1 .**

Find the mean value of dataset *X* using (1) on each variable (*x*, *y*, *p*):

(1) $$\begin{array}{c} \overset{-}{X}=\frac{\sum_{i=1}^{n}Xi}{N}, \end{array}$$

where *N* is the number of available samples.

**Step 2 .**

Subtract the mean value ($\overset{-}{X})$ from each sample value (*X*) as shown in the following equation to have a new matrix (dataadjust) with the same dimension, *M*(*N*∗*M*):

(2) $$\begin{array}{c} \Phi i=Xi-\overset{-}{X}. \end{array}$$

**Step 3 .**

Compute the covariance of any two variables, (*x*, *y*), (*x*, *p*), and (*y*, *p*), separately using (3) on the previous matrix (*N*∗*M*):

(3) $$\begin{array}{c} \text{Cov}\left(M\right)=\frac{\sum_{i=1}^{n}\left(Xi-\overset{-}{X}\right)\left(Yi-\overset{-}{Y}\right)}{\left(N-1\right)}. \end{array}$$

**Step 4 .**

Using the following equation, compute the eigenvalues from covariance matrix:

(4) $$\begin{array}{c} \left|M-\lambda I\right|=0. \end{array}$$

**Step 5 .**

Also, calculate the eigenvectors from the covariance matrix using the following equation:

(5) $$\begin{array}{c} \left(M-\lambda jI\right)ej=0. \end{array}$$

**Step 6 .**

Finally, retain the largest eigenvectors *K* as the principal components with respect to the eigenvalues.

Since we exploited MATLAB workstation for our implementation, hence, we provide some insight on the conversion of some terminologies such as loading to latent, eigenvalue to score, and eigenvector to component. The latent is a vector describing all the observations in a signature. For each latent, we calculate the projection error to get the score value with respect to its latent. Finally, the component is a combination of three elements, and it is calculated as follows:

(6) $$\begin{array}{c} \text{component}=\text{score}\times \text{latent}+\text{residual}. \end{array}$$

After PCA transforms the data, the result obtained is composed of three components as features because our dataset space is three dimensional with *x*, *y*, and *p* variables. We could reconstruct the original data by these components. The information that is not going to be explained by the components in original data is called the residual. The number of components is dependent on the value of the residual information.

Therefore, any of the three resulting components can be used to represent the original signature observations. The values in the score matrix are ranked based on their variance in a decreasing order, which also corresponds to the arrangement of the principal components. For instance, the first component has the highest variance value with respect to its score compared to the other two components. Likewise, the second component has the second highest variance while the third component has the least variance value.

### 3.2. Verification

The classifier used in this experiment is MLP neural network, which is based on a supervised learning technique called backpropagation. Basically, a MLP neural network is composed of an input layer, a hidden layer, and an output layer, which also corresponds to the flow of distribution of the feature vector in the network to attain a desired output. The computation of neural network involves a set of input signals, synaptic weight at each neuron, and a bias. The output is some function of weighted summation of the input. This function is the activation function, which maps the amplitude of values of the output into a certain range. The training of the network is an iterative procedure. In each iteration, weight coefficients (*w*) of neurons are changed based on the output error that is propagated from the output layer to the front layer to estimate the hidden layer errors [27].

In the beginning of training, the weights (*w*) are initialized with small values between 0 and 1 and the output of each neuron is an input for feeding the next hidden layer [23]. In the paragraph below, the learning procedure in backpropagation network is explained.

The output (*y*) is linear combinations of inputs and can be computed, where *i* is index of input, *l* is index of neuron, and *N* is the number of input samples [28], as follows:

(7) $$\begin{array}{c} Y=\overset{N}{\underset{l=1}{\sum }}w_{il}x_{l}+w_{in+1}. \end{array}$$

Then, the output (*y*) is compared with the desired output, resulting in an error (*e*). The following equation shows how error is calculated, where *tl* are the target values and *ol* are the output values [28]:

(8) $$\begin{array}{c} E=\frac{1}{2}\overset{N}{\underset{l=1}{\sum }}{\left(tl-ol\right)}^{2}. \end{array}$$

As a result, the error (*e*) for each neuron is used for adjusting the weight, with the aim of attaining the desired output; the error sends back to find the error value (*δ*) of each layer (e.g., layer *j*) in lower hidden layers based on its higher layer (*K*) error as follows:

(9) $$\begin{array}{c} \delta j=oj\left(1-oj\right)\underset{k}{\sum }w_{kj}\delta_{k}. \end{array}$$

Finally, the error in each neuron is used to update the neuron weights in order to minimize the total error value to achieve an output value close to the desired output. It can be calculated using the following equation, where *η* is learning rate:

(10) $$\begin{array}{c} w_{ij}^{k+1}=w_{ij}+\eta \delta_{j}o_{i}. \end{array}$$

## 4. Experimental Result

In this paper, we used ten genuine signature samples and five skill-forged signature samples for each user. In addition, we included another five genuine signature samples from a randomly selected user (user 193) to have non-skill-forged signatures. Similarly, in the testing phase, another ten genuine signature samples of the same user and five skill-forged signature samples of that user and five genuine signature samples from user 193 are combined to make the testing matrix.

We here note that the selection of principal components for attaining a reliable recognition rate is quite heuristic. Therefore, we initially utilized all the three achieved components as features. As a result, the feature vector is composed of only nine values rather than the high-dimension space to represent a signature sample. According to our experimental result, these nine features are not enough to model a reliable online signature verification system, as the recognition rate was only 82%.

**Figure 4:**
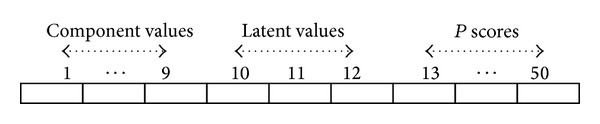
A vector to represent a user's signature.

Afterwards, we resorted to exploring the proposed PCA feature selection strategy, which consists of other information, such as latent and score as explained in Section 3. In addition to the nine features used in the previous experiment, the first latent vector from the first component is selected. Also, we utilized 38 score values from the whole score matrix. Therefore, the resulting feature representation for each signature consisted of 50 prominent features that are combinations of nine component values, three latent values, and thirty eight scores, as shown in Figure 4.

**Figure 5:**
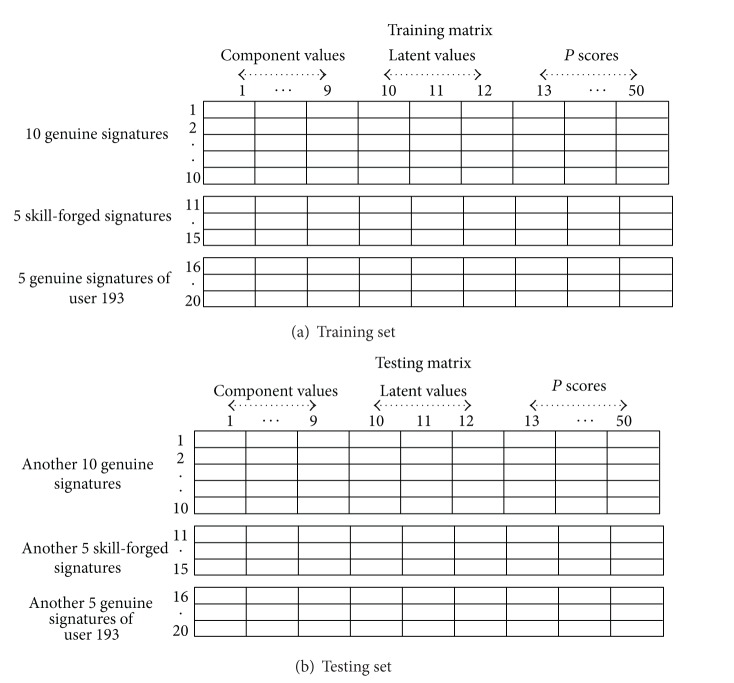
Sample of training and testing matrices per user.

However, the length of each signature varies from that of another; as such, we used a random score value selection instead of selecting the first 38 score values from the score matrix, since we have different length for each score matrix in any signature sample. Furthermore, we used a variable *q* as a sampling step, which is a selector that is defined by the following decisions, shown in Table 2. For example, if the size of our score matrix for the first signature sample is more than 190, the *q* step is defined as 5.  Figure 5 shows a schema of user signatures' training and testing matrices with 20 samples for each user.

After dividing the mentioned subset into training and testing data, the desired output for both training and testing data should be identified as well to evaluate the performance of the system through supervised learning. Since the dimension of training and testing data is 20 × 50, the desired output matrix dimension is supposed to be two 20 × 1 matrices. The value 1 indicates the genuine class, and 0 denotes the forged class based on the sigmoid function that is selected for the output layer.

Table 3 summarizes the architecture of a two-layer feed-forward neural network with 50 neurons in the input layer, 20 neurons in the hidden layer, and 1 neuron in the output layer. The Levenberg-Marquardt optimization technique, which has the lowest error, is used for achieving the optimum adjusted weight values. For calculating the performance, mean square error (MSE) is used.

Table 4 shows the test results, where the proposed technique is able to achieve 7.4% and 6.4% for the false acceptance rate (FAR) and false rejection rate (FRR), respectively, and 93.1% accuracy as calculated using the following equation:

(11) $$\begin{array}{c} \text{Accuracy}\left(\%\right)=100\left(\%\right)-\left[\frac{\left(\text{FRR}+\text{FAR}\right)}{2}\right]. \end{array}$$

**Figure 6:**
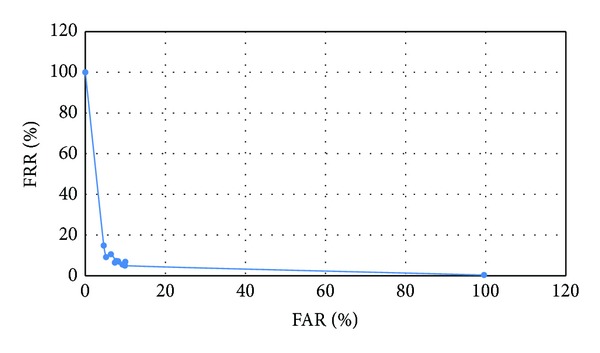
ROC curve of the proposed model.

For evaluating the proposed OSV model, receiver operating characteristic (ROC) curve is used. The ROC curve shows the visual plot of FAR against FRR based on variety of thresholds between 0 and 1. The optimum threshold should minimize the false negative and false positive values. The ROC curve for the proposed technique is shown in Figure 6. The result shows that the optimum threshold value is 0.4.

To gain a better understanding of the effect of the selected features from PCA analysis on recognition results, a comparison of previous approaches on the SIGMA database is shown in Table 5. The comparison shows that the proposed feature selection method which resulted in 50 subset features is more efficient than the previous methods. Meanwhile, it is obvious from Table 5 that despite using similar classifier (ANN), the same number of samples for training and testing, but different feature selection and extraction strategies, the proposed method outperformed the techniques presented in [14, 15]. With regard to this, we denote that not only could the PCA coefficients be effective as features in verification but also the latent and score can serve as additional features in attaining higher accuracy.

## 5. Discussion 

This study measured the performance of the proposed OSV system based on 50 selected features after implementing PCA on the signature to represent it in the verification system. Moreover, 200 users with 8,000 signature samples have been used in this study to estimate the recognition accuracy, which is 93.1%. It is also obvious that a smaller number of signature features in the training phase caused the results to have less validity, achieving more FAR and FRR and less accuracy.

In addition, the result attained in this experiment shows that not only can the components (as features) retrieved from principal component analysis, which has been commonly adopted in the previous studies, be utilized in online and offline signature verification, but also other elements, such as latent and score values, could be used to achieve a high accuracy rate.

As shown in Table 4, the FRR and FAR obtained are nearly equal. This nearly equivalent value means that the errors to detect the genuine and forged signatures are almost the same. Based on this fact, the average of FAR and FRR is defined as a misclassified rate, with 6.9% that is approximately close to an equal error rate (EER). Nevertheless, the length of the signature sample is considered to be more than 38 pen trajectories (*x*, *y*) and pressure samples (*p*) to compute *q* value from score element, where the minimum signature length of the signature in this study was more than 100 observations.

## 6. Conclusion

A new approach for feature selection in verification and recognition of online handwritten signatures is presented in this paper. Utilizing PCA for feature extraction on Malaysian handwritten signatures, we proposed to extract 50 prominent features to represent each individual signature. Afterwards, a MLP is implemented to classify the signatures as either forged or genuine. The verification result shows the effectiveness of the proposed technique, as it attained 93.1% accuracy on 200 users and 8,000 signatures consisting of genuine and skill-forged signatures.

## Figures and Tables

**Table 1:** Number of samples per user in training and testing.

Genuine signature samples	Skill-forged signature samples	Non-skill-forged samples	Number of users	Total samples
20	10	10	200	8000

**Table 2:** Step value of choosing (*q*).

Size of score matrix	Step value
≥190	5
≥152	4
≥114	3
≥76	2
<76	1

**Table 3:** Neural network architecture.

Type	Training algorithm	Activation function	Performance function	Number∗
MLP	Levenberg-Marquardt	Sigmoid	MSE	50 20 1

*Number: number of neurons in input, hidden, and output layers.

**Table 4:** Recognition and error rates.

Accuracy (%)	FAR (%)	FRR (%)
93.1	7.4	6.4

**Table 5:** Some related works on SIGMA database.

References	Classifier	Feature extraction	No. of obtained features	No. of samples in training	No. of samples in testing	FAR (%)	FRR (%)	Accuracy rate (%)	Threshold value
Iranmanesh et al. [14]	ANN	Pearson Correlation	9	4000	4000	21.3	13.8	82.4	N/A
Malallah et al. [15]	ANN	PCA	162	4000	4000	8.5	24.3	83.5	N/A
Proposed technique	ANN	PCA	50	4000	4000	7.4	6.4	93.1	0.4
